# Validation of reference genes for expression analysis in a murine trauma model combining traumatic brain injury and femoral fracture

**DOI:** 10.1038/s41598-020-71895-x

**Published:** 2020-09-14

**Authors:** Ellen Otto, Paul Köhli, Jessika Appelt, Stefanie Menzel, Melanie Fuchs, Alina Bahn, Frank Graef, Georg N. Duda, Serafeim Tsitsilonis, Johannes Keller, Denise Jahn

**Affiliations:** 1 Julius Wolff Institute for Biomechanics and Musculoskeletal Regeneration, Charité-Universitätsmedizin Berlin, 13353 Berlin, Germany; 2 Center for Musculoskeletal Surgery, Charité-Universitätsmedizin Berlin, 13353 Berlin, Germany; 3 Department of Trauma and Orthopedic Surgery, University Medical Center Hamburg-Eppendorf, 20246 Hamburg, Germany

**Keywords:** Biological techniques, Molecular biology, Medical research, Molecular medicine

## Abstract

Systemic and local posttraumatic responses are often monitored on mRNA expression level using quantitative real-time PCR (qRT-PCR), which requires normalisation to adjust for confounding sources of variability. Normalisation requests reference (housekeeping) genes stable throughout time and divergent experimental conditions in the tissue of interest, which are crucial for a reliable and reproducible gene expression analysis. Although previous animal studies analysed reference genes following isolated trauma, this multiple-trauma gene expression analysis provides a notable study analysing reference genes in primarily affected (i.e. bone/fracture callus and hypothalamus) and secondarily affected organs (i.e. white adipose tissue, liver, muscle and spleen), following experimental long bone fracture and traumatic brain injury. We considered tissue-specific and commonly used top-ranked reference candidates from different functional groups that were evaluated applying the established expression stability analysis tools NormFinder, GeNorm, BestKeeper and RefFinder. In conclusion, reference gene expression in primary organs is highly time point as well as tissue-specific, and therefore requires careful evaluation for qRT-PCR analysis. Furthermore, the general application of *Ppia*, particularly in combination with a second reference gene, is strongly recommended for the analysis of systemic effects in the case of indirect trauma affecting secondary organs through local and systemic pathophysiological responses.

## Introduction

Although pre-hospital and in-hospital care has profoundly advanced over the past decades^1,2^, major trauma remains a leading cause of death and disability, mainly in younger ages^3,4^. Treatment of patients with multiple injuries presents a medical challenge, as the complex posttraumatic response is injury-unique and includes the interaction between different organ systems^5–7^. Despite strenuous clinical and experimental efforts^8–11^, the underlying pathophysiological and immunological responses triggered by the combination of multiple injuries still remain to be elucidated.

Systemic and local posttraumatic gene response is commonly monitored on mRNA expression level^12–15^, using the cost-efficient, easy to adapt and widely established method of quantitative real-time reverse transcription-polymerase chain reaction (qRT-PCR). Despite its advantages, the results are influenced by confounding sources of variability during mRNA extraction, quantification and cDNA transcription. Thus, internal normalisation through a reference gene is required for accurate data analysis^16^. The ideal reference or housekeeping gene for normalisation is stable in the tissue of interest, throughout time and under all experimental conditions. However, previous studies demonstrated that commonly used reference genes such as *Gapdh* are indeed differentially regulated among distinct cell types and organ systems^17–19^. Therefore, the time-consuming identification of suitable housekeeping genes for the qRT-PCR data normalisation remains essential for each newly designed study, in order to allow an accurate analysis. Although first attempts have been made to recommend reference genes for qRT-PCR analysis in isolated trauma models^20–22^, there are currently no multiple-injury studies determining suitable reference genes.

This study aimed to identify suitable reference genes in primarily and secondarily affected organs following experimental long bone fracture (Fx) and traumatic brain injury (TBI). We considered the commonly used and tissue-specific top-ranked reference candidates from different functional groups (Table 1): *Actb* (*Actin beta*), *B2M* (*Beta-2-Microglobulin*), *Gapdh* (*Glyceraldehyde-3-Phosphate Dehydrogenase*), *Hmbs* (*Hydroxymethylbilane Synthase*), *Hprt* (*Hypoxanthine Phospho-ribosyltransferase 1*), *Ppia* (*Peptidylprolyl Isomerase A or Cyclophilin A*), *Psmb2* (*Proteasome Subunit Beta 2*), *Rplp0* (*Ribosomal Protein Lateral Stalk Subunit P0*), *Srsf4* (*Serine And Arginine Rich Splicing Factor 4*) and *Tbp* (*TATA-Box Binding Protein*). All candidate genes were evaluated by applying the established expression stability analysis tools NormFinder, GeNorm, BestKeeper and RefFinder. To our knowledge, this extensive gene expression analysis is the first multiple-trauma study analysing reference genes in both primary and secondary organs, which provides useful guidelines for future gene expression analyses.

**Table 1:** qPCR primer set (murine) of all reference genes tested, contrasting the top-ranked generally applied (bold = applied in all tissues) and the tissue-specific (unbold = applied in selected tissues) candidates.

Symbol	Gene name	Protein function	Primer sequence (F: 5′-3′/R: 3′-5′)
***Actb***	**Actin beta**	**Structural protein of the cytoskeleton, involved in cell structure, integrity, motility and intercellular signalling**	**F: TTGCTGACAGGATGCAGAAG** **R: GTACTTGCGCTCAGGAGGAG**
***B2m***	**Beta-2-microglobulin**	**Component of the class I major histocompatibility complex (MHC), involved in the presentation of peptide antigens to the immune system**	**F: TCTCACTGACCGGCCTGTAT** **R: GATTTCAATGTGAGGCGGGTG**
***Gapdh***	**Glyceraldehyde-3-phosphate dehydrogenase**	**Catalyses of the reversible oxidative phosphorylation in glycolysis and gluconeogenesis; participates in nuclear events incl. transcription, RNA transport, DNA replication and apoptosis**	**F: ACTGAGCAAGAGAGGCCCTA** **R: TATGGGGGTCTGGGATGGAA**
***Hprt***	**Hypoxanthine phospho-ribosyltransferase 1**	**Transferase that catalyses a central reaction in the synthesis of purine nucleotides**	**F: GGTTAAGCAGTACAGCCCCA** **R: GGCCTGTATCCAACACTTCG**
***Ppia***	**Peptidylprolyl Isomerase A or Cyclophilin A**	**Catalyses the cis–trans isomerisation of proline imidic peptide bonds in oligopeptides and accelerates the protein folding**	**F: CCACCGTGTTCTTCGACATC** **R: CTGGCACATGAATCCTGGAA**
*Hmbs*	Hydroxymethylbilane synthase	Third enzyme of the haem biosynthetic pathway	F: ATGAGGGTGATTCGAGTGGG R: TTGTCTCCCGTGGTGGACATA
*Psmb2*	Proteasome subunit Beta 2	Component of the 20S core proteasome complex, involved in the proteolytic degradation of most intracellular proteins	F: ATGTGTTGGAGAGGCTGGAG R: AAGTTAGCTGCTGCTGTGGG
*Rplp0*	Ribosomal protein lateral stalk subunit P0	A component of the large 60S subunit of the ribosome	F: TTTGACAACGGCAGCATTTA R: GTACCCGATCTGCAGACACAC
*Srsf4*	Serine and arginine rich splicing factor 4	Involved in mRNA processing	F: TTGTGGAGAATTTGTCAAGTCG R: GTTTTTGCGTCCCTTGTGAG
*Tbp*	TATA-box binding protein	General transcription factor, involved in the initial transcriptional step of the pre-initiation complex followed by RNA polymerase II activation	F: CCAGAACAACAGCCTTCCAC R: GTGGAGTAAGTCCTGTGCCG

## Results

We determined the most suitable reference genes in major organ systems of our murine trauma model, which combines long bone fracture (Fx) and traumatic brain injury (TBI), at three (d3) and seven days (d7) after injury. Besides evaluating the reference genes within the primary organs of the trauma model (i.e. bone/fracture callus and hypothalamus), we additionally investigated the secondary organs (i.e. white adipose tissue, liver, muscle and spleen), which are highly metabolic- and immuno-active following trauma^23–26^.

Our gene expression stability analysis addressed tissue-specific and commonly used reference genes. Once the threshold cycle (Ct) was determined, each candidate reference gene was evaluated applying NormFinder, GeNorm, BestKeeper and validated using RefFinder. While the NormFinder algorithm calculates the stability value (sv) according to the inter- and intra-group variation, GeNorm defines the reference gene stability value (M) through pairwise variation, both aiming for the lowest value representing the highest expression stability. BestKeeper generates the coefficient of correlation (r) and a standard deviation (SD) of the crossing point (Cp) which is analogue to the Ct. Finally, we summarised all individual ranks identified in the final rank. To verify our results, the RefFinder algorithm was applied, combining the comparative delta-Ct method, NormFinder, GeNorm and BestKeeper.

### Bone/fracture callus analysis

The expression analysis in primarily traumatised organs is challenging, as the gene expression pattern fundamentally changes. In our fracture model, we aimed to compare the intact bone with fracture callus tissue, both consisting of highly different cell composition. As the callus in the applied model offers low sample volume, only five predominantly applied reference genes were tested.

**Figure 1:**
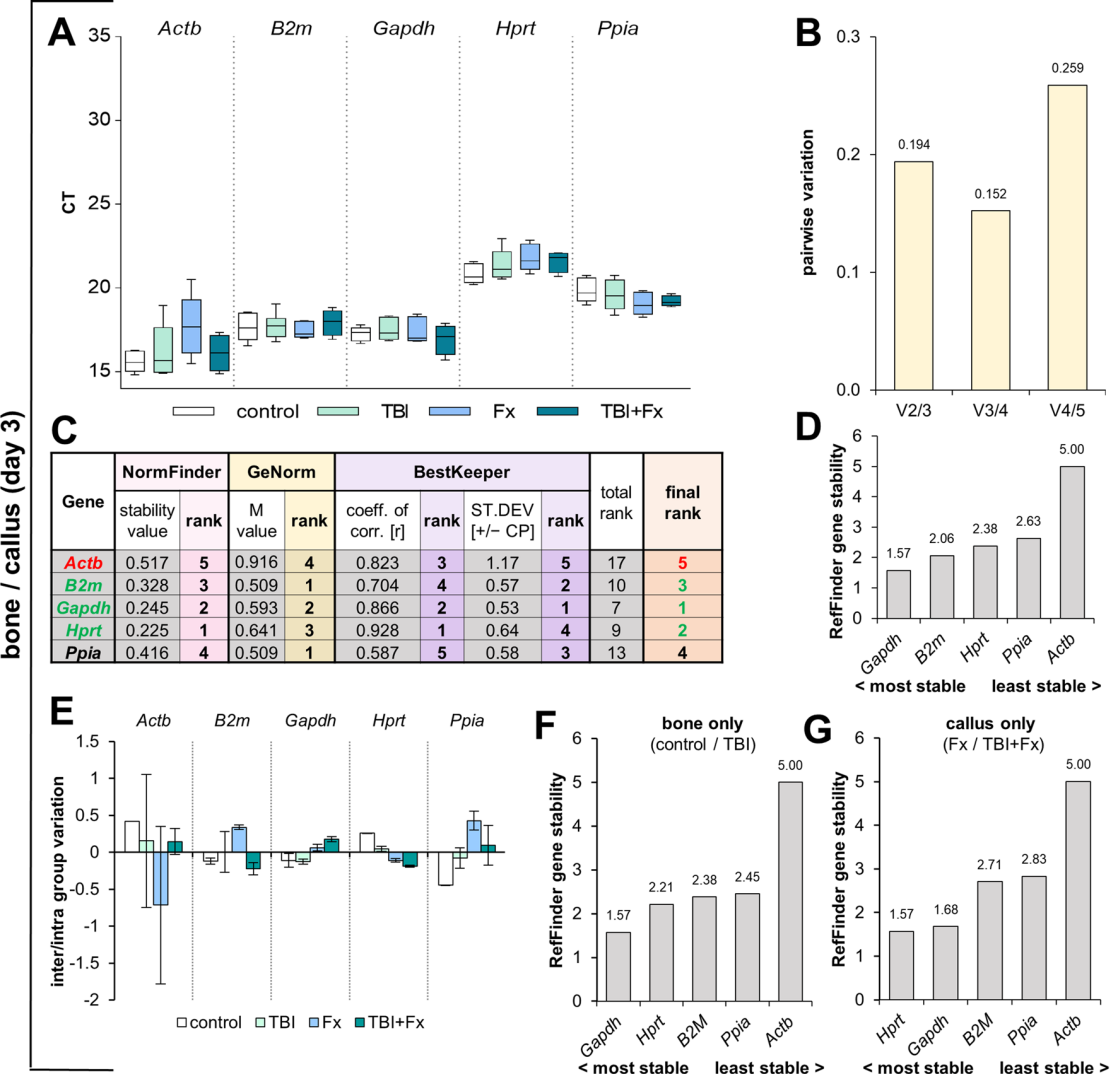
Reference gene expression stability analysis in bone/callus tissue at 3 days following surgery. (**A**) Distribution of threshold cycle (Ct) values for each group (Fx = fracture; TBI = traumatic brain injury; TBI + Fx = combined injury) of all candidate reference genes tested. (**B**) Pairwise variation (V_n/n+1_) analysis by GeNorm, to determine the optimal number of reference genes for normalisation. (**C**) Stability analysis by NormFinder (stability value: sv), GeNorm (M value) and BestKeeper (coefficient of correlation: r, standard deviation: SD), with a sum displayed in the final rank (green = most stable, red = least stable). (**D**) Overall expression stability confirmation of RefFinder, which combines NormFinder, GeNorm, BestKeeper and the comparative delta-Ct method. (**E**) inter- and intra-group variation calculated with NormFinder. (**F/G**) expression stability confirmation of RefFinder for intact bone (**F**) or callus tissue (**G**).

At d3 after trauma in bone/callus, we observed a strong expression (low Ct) of all reference genes tested (Fig. 1A). While NormFinder ranked *Hprt* (sv = 0.225) first, *Gapdh* (sv = 0.245) second, and *Actb* (sv = 0.517) last (Fig. 1C), GeNorm identified the highest stability for *B2m* and *Ppia* (M = 0.509), but also *Actb* (M = 0.916) as the least stable. As none of the candidate reference genes reached the threshold for homogeneous sample panels (M < 0.5) during the GeNorm analysis, the standard threshold for heterogeneous samples (M < 1.5) was applied^27,28^. GeNorm’s pairwise variation revealed the need for the combination of three reference genes (V_3/4_ = 0.15) (Fig. 1B). The BestKeeper algorithm calculated a high coefficient of correlation for *Hprt* (r = 0.928) and *Gapdh* (r = 0.866) and low standard deviations for *Gapdh* (SD = 0.53) and *B2m* (SD = 0.57). Our final ranking, as a summation of the single ranks, determined *Gapdh*, *Hprt* and *B2m* as the most suitable candidates for a combined bone and fracture callus analysis at three days after trauma (Fig. 1C). This combination was also verified by RefFinder (Fig. 1D). An additional inter- and intra-group variation analysis identified a strong dysregulation of *Actb* following trauma (Fig. 1E), which was confirmed by the separate evaluation of reference genes in the intact bone or in the fracture callus using RefFinder. In both tissues bone and callus, *Gapdh*, *Hprt* and *B2m* remained the most stable reference genes, while *Actb* showed the strongest variability (Fig. 1F,G).

**Figure 2:**
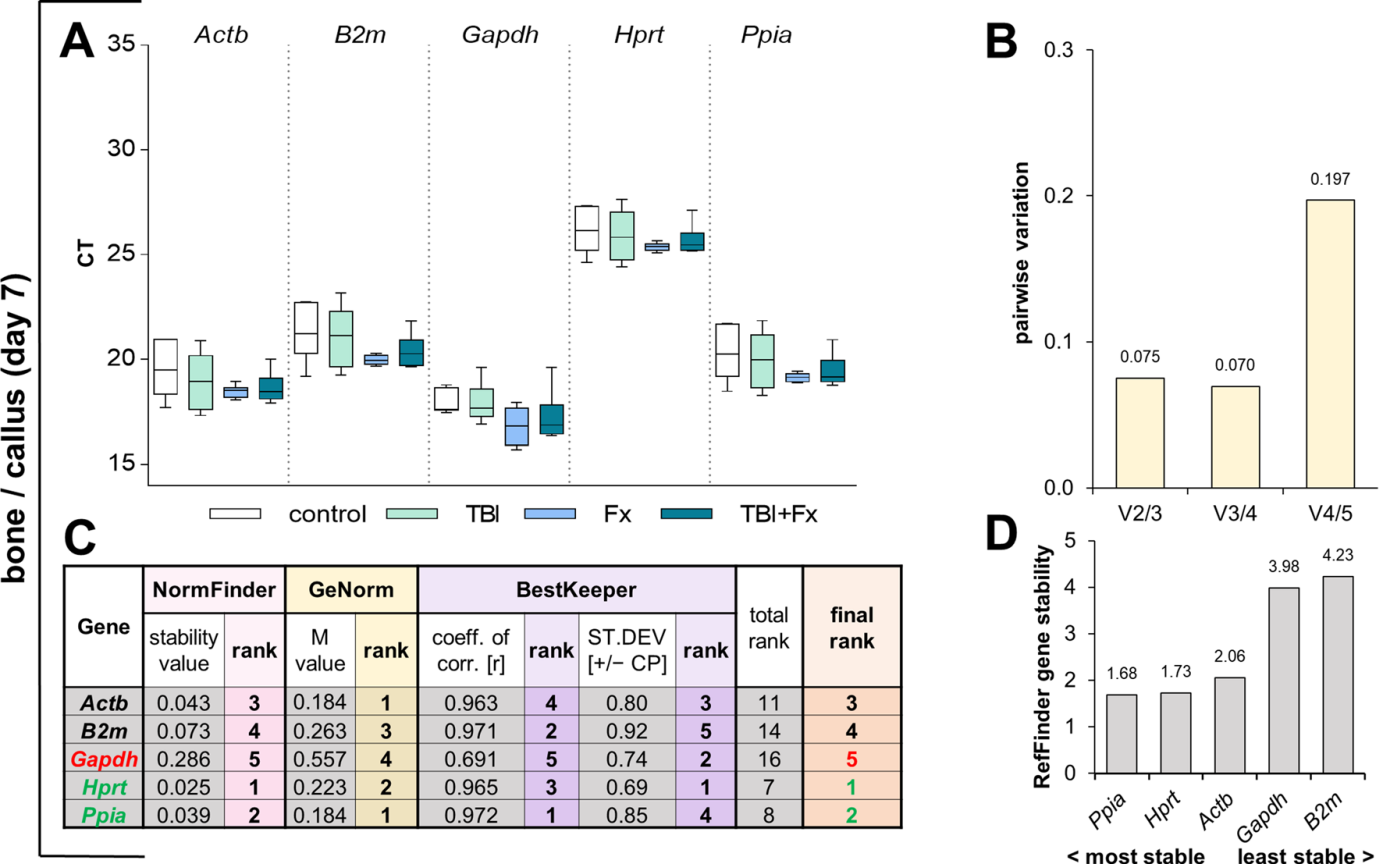
Reference gene expression stability analysis in bone/callus tissue at seven days following surgery. (**A**) Distribution of threshold cycle (Ct) values for each group (Fx = fracture; TBI = traumatic brain injury; TBI + Fx = combined injury) of all candidate reference genes tested. (**B**) Pairwise variation (V_n/n+1_) analysis by GeNorm, to determine the optimal number of reference genes for normalisation. (**C**) Stability analysis by NormFinder (stability value: sv), GeNorm (M value) and BestKeeper (coefficient of correlation: r, standard deviation: SD), with a sum displayed in the final rank (green = most stable, red = least stable). (**D**) Overall expression stability confirmation of RefFinder, which combines NormFinder, GeNorm, BestKeeper and the comparative delta-Ct method.

At d7 after trauma in bone/callus, we conducted the same evaluation as for d3, as the healing process provokes a great gene expression modulation in the fracture callus, as well as in intact bone (Fig. 2A–D). Here NormFinder ranked *Hprt* first and *Ppia* second (Fig. 2C), whereas GeNorm calculated the highest stability for *Actb* and *Ppia*. The GeNorm analysis additionally revealed that the combination of two reference genes is sufficient for housekeeping in bone and callus tissue at d7 after trauma (Fig. 2B). BestKeeper calculated the highest coefficient of correlation for *Ppia* and the lowest SD for *Hprt*. Our final ranking and RefFinder confirmed *Ppia* and *Hprt* as the most suitable reference genes in bone and fracture callus seven days after trauma (Fig. 2C,D).

### Hypothalamus analysis

Analogue to the fracture callus, gene expression in the primarily traumatised brain following TBI fundamentally changed, especially regarding inflammatory processes. Neuroinflammation was confirmed and monitored with established TBI-related immune markers^29,30^ (for selection see Supplementary Fig. S1 online). Like the fracture callus, the hypothalamus offered a low sample volume, which again allowed the testing of only the five predominantly applied reference genes.

**Figure 3:**
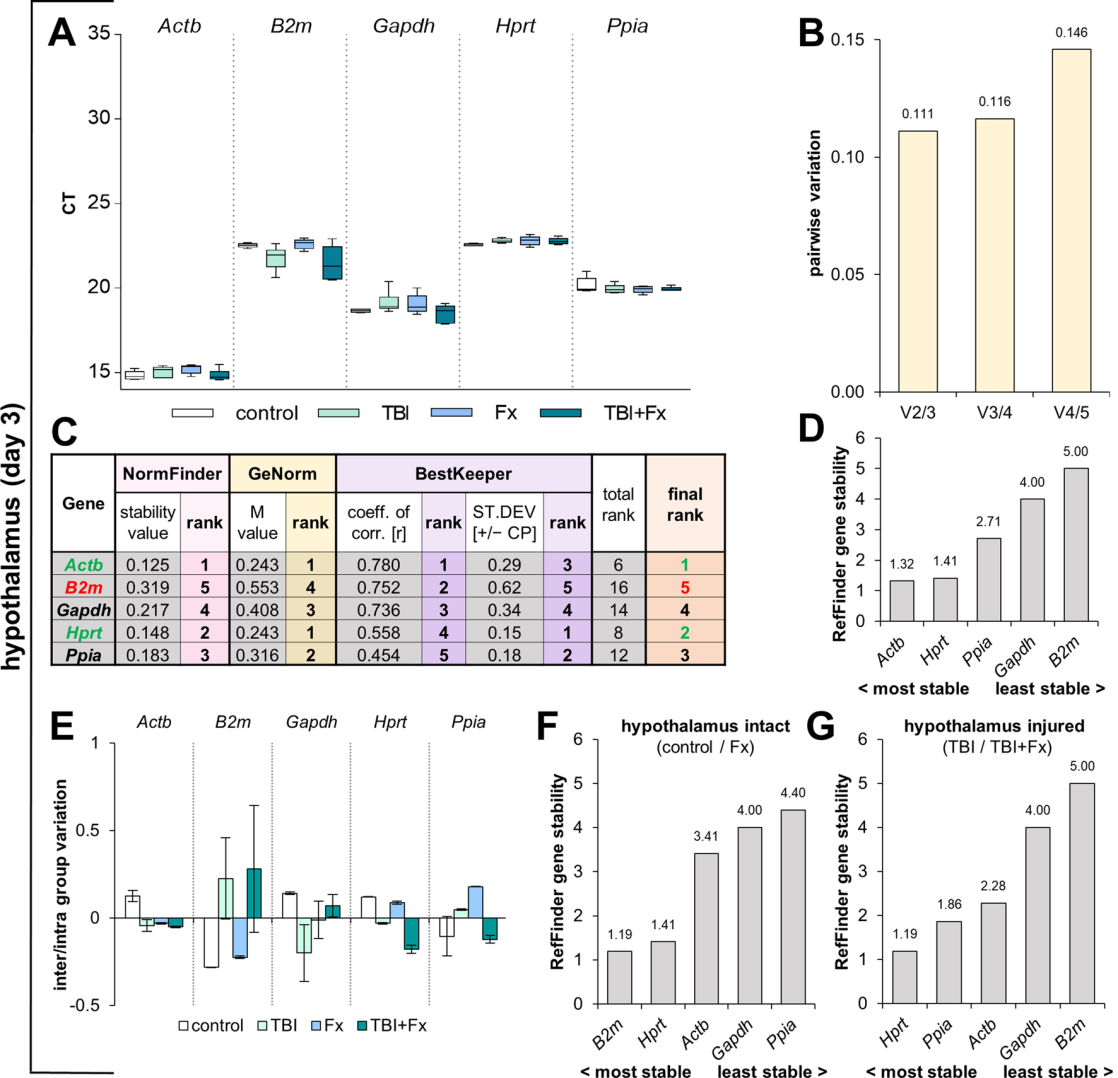
Reference gene expression stability analysis in hypothalamus tissue at three days following surgery. (**A**) Distribution of threshold cycle (Ct) values for each group (Fx = fracture; TBI = traumatic brain injury; TBI + Fx = combined injury) of all candidate reference genes tested. (**B**) Pairwise variation (V_n/n+1_) analysis by GeNorm, to determine the optimal number of reference genes for normalisation. (**C**) Stability analysis by NormFinder (stability value: sv), GeNorm (M value) and BestKeeper (coefficient of correlation: r, standard deviation: SD), with a sum displayed in the final rank (green = most stable, red = least stable). (**D**) Overall expression stability confirmation of RefFinder, which combines NormFinder, GeNorm, BestKeeper and the comparative delta-Ct method. **(E)** inter- and intra-group variation calculated with NormFinder. **(F/G)** expression stability confirmation of RefFinder for intact hypothalamus **(F)** or injured hypothalamus **(G)**.

At d3 after trauma in the hypothalamus, we observed a strong expression (low Ct) of all reference genes tested (Fig. 3A). NormFinder and GeNorm identified *Actb* (sv = 0.125, M = 0.243) and *Hprt* (sv = 0.148, M = 0.243) as the most stable reference genes (Fig. 3C). The GeNorm algorithm revealed that the combination of two genes (V_2/3_ = 0.111) is sufficient as a reference in the hypothalamus at d3 after trauma (Fig. 3B). BestKeeper calculated the highest coefficient of correlation for *Actb* (r = 0.780) and the lowest SD for *Hprt* (SD = 0.15). The results of NormFinder, GeNorm, BestKeeper and the final rank identified *Actb* and *Hprt* as the most suitable combination of housekeepers (Fig. 3C), which was confirmed by RefFinder (Fig. 3D). All algorithms defined *B2m* as the least suitable reference gene, which exclusively missed the GeNorm threshold. An additional inter- and intra-group variation analysis revealed a strong regulation of *B2m* in the injured hypothalamus in contrast to the intact hypothalamus (Fig. 3E). Although *B2m* is highly regulated in the injured hypothalamus (Fig. 3G), it remained the most stable housekeeper in the intact hypothalamus (Fig. 3F).

**Figure 4:**
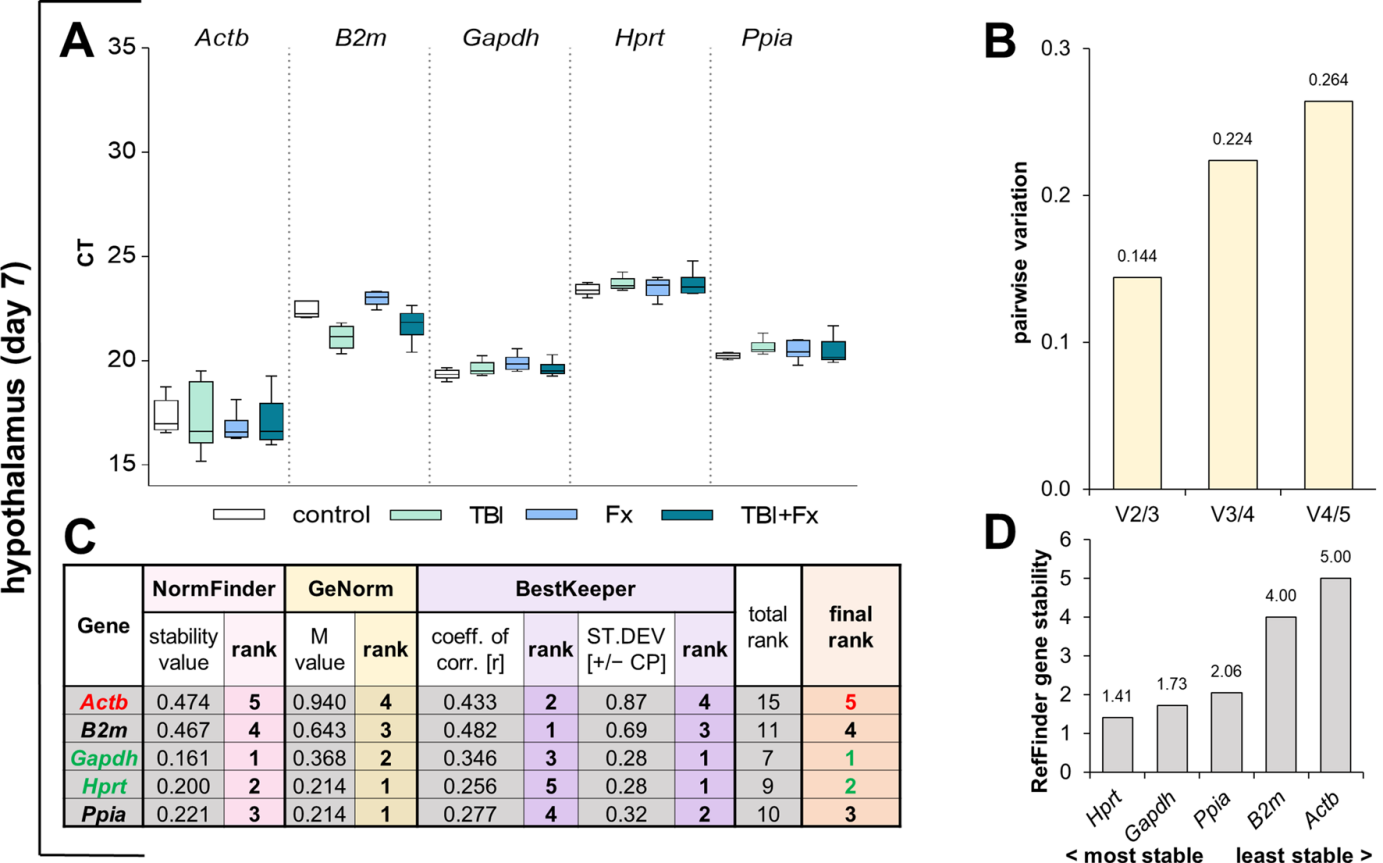
Reference gene expression stability analysis in hypothalamus tissue at seven days following surgery. (**A**) Distribution of threshold cycle (Ct) values for each group (Fx = fracture; TBI = traumatic brain injury; TBI + Fx = combined injury) of all candidate reference genes tested. (**B**) Pairwise variation (V_n/n+1_) analysis by GeNorm, to determine the optimal number of reference genes for normalisation. (**C**) Stability analysis by NormFinder (stability value: sv), GeNorm (M value) and BestKeeper (coefficient of correlation: r, standard deviation: SD), with a sum displayed in the final rank (green = most stable, red = least stable). (**D**) Overall expression stability confirmation of RefFinder, which combines NormFinder, GeNorm, BestKeeper and the comparative delta-Ct method.

At d7 after trauma in the hypothalamus, we observed a strong expression (low Ct) of all reference genes tested (Fig. 4A). NormFinder determined the best stability for *Gapdh*, while GeNorm identified *Hprt* and *Ppia* as the most adequate reference gene combination (Fig. 4C). During the GeNorm analysis *Actb* and *B2m* failed the suitability threshold. GeNorm revealed that the combination of two reference genes is sufficient in the hypothalamus at d7 (Fig. 4B). BestKeeper calculated the lowest SD for *Gapdh* and *Hprt*. Our final ranking and RefFinder confirmed *Gapdh* and *Hprt* as the most suitable reference genes in hypothalamus at d7 after trauma (Fig. 4C,D).

### White adipose tissue (WAT) analysis

**Figure 5:**
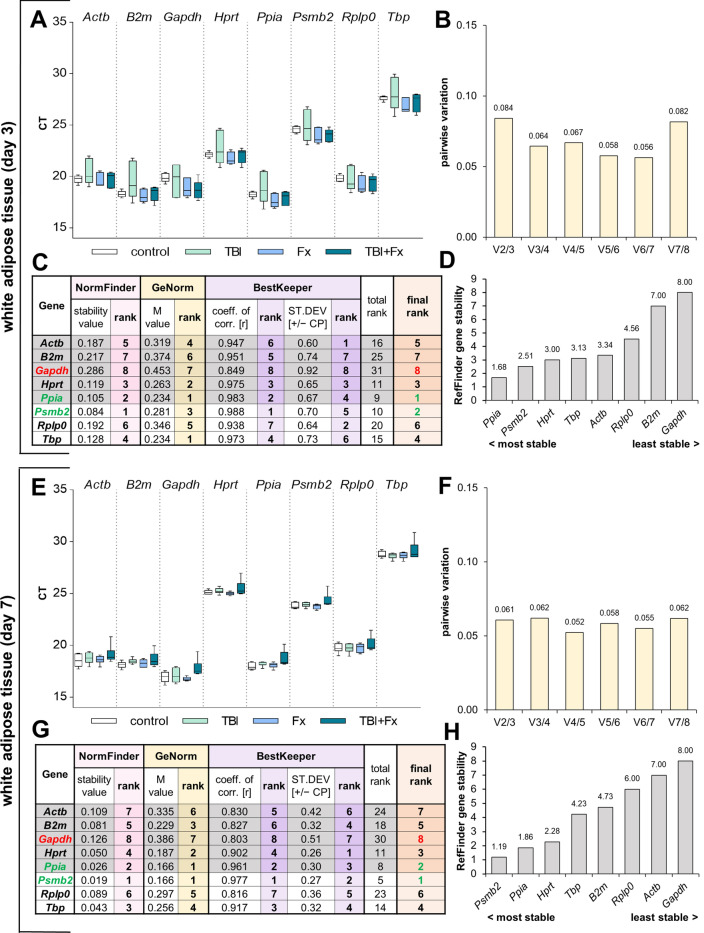
Reference gene expression stability analysis in white adipose tissue (WAT) at three (**A**–**D**) and seven days (**E**–**H**) following surgery. (**A**/**E**) Distribution of threshold cycle (Ct) values for each group (Fx = fracture; TBI = traumatic brain injury; TBI + Fx = combined injury) of all candidate reference genes tested. (**B**/**F**) Pairwise variation (V_n/n+1_) analysis by GeNorm, to determine the optimal number of reference genes for normalisation. (**C**/**G**) Stability analysis by NormFinder (stability value: sv), GeNorm (M value) and BestKeeper (coefficient of correlation: r, standard deviation: SD), with a sum displayed in the final rank (green = most stable, red = least stable). (**D**/**H**) Overall expression stability confirmation of RefFinder, which combines NormFinder, GeNorm, BestKeeper and the comparative delta-Ct method.

As metabolism is strongly regulated after trauma, we investigated the stability of recommended reference genes in WAT at d3. Here gene expression was highest (low Ct) for *Ppia* and lowest for *Tbp* (Fig. 5A). NormFinder ranked *Psmb2* (sv = 0.084) the best target gene and *Ppia* (sv = 0.105) second. Highest variation was observed for *Gapdh* (sv = 0.286) (Fig. 5C). GeNorm revealed the best M value for *Ppia* and *Tbp* (M = 0.234), as well as a highly variable regulation of *Gapdh* (M = 0.453). During the GeNorm analysis all investigated housekeepers reached the threshold of suitability of M < 0.5, while showing a sufficiency for the application of two reference genes (V_2/3_ < 0.15) in WAT d3 after trauma (Fig. 5B). The BestKeeper algorithm determined the highest correlation for *Psmb2* (r = 0.988) and *Ppia* (r = 0.983). In contrast, *Gapdh* was the least eligible housekeeping gene tested with a low coefficient of correlation and high SD (Fig. 5C). Overall, our final ranking identified *Ppia* and *Psmb2* as the most stable reference genes in WAT three days after trauma (Fig. 5C). RefFinder verified *Ppia* and *Psmb2* as the best combination, with *Gapdh* showing again the strongest differential regulation (Fig. 5D).

To prove that the suggested combination of *Ppia* and *Psmb2* from d3 remains suitable even at seven days after trauma, we conducted the same evaluation for d7 (Fig. 5E-H). P*pia* and *Psmb2* showed the best outcome among all algorithms applied, while *Gapdh* remained the most variable. In GeNorm all tested genes reached the threshold, and the algorithm revealed that two housekeeping genes are sufficient for a subsequent gene expression analysis (Fig. 5F). Our final rank and RefFinder confirmed *Ppia* and *Psmb2* as the best reference genes in white adipose tissue at seven days after trauma (Fig. 5H).

### Liver analysis

**Figure 6:**
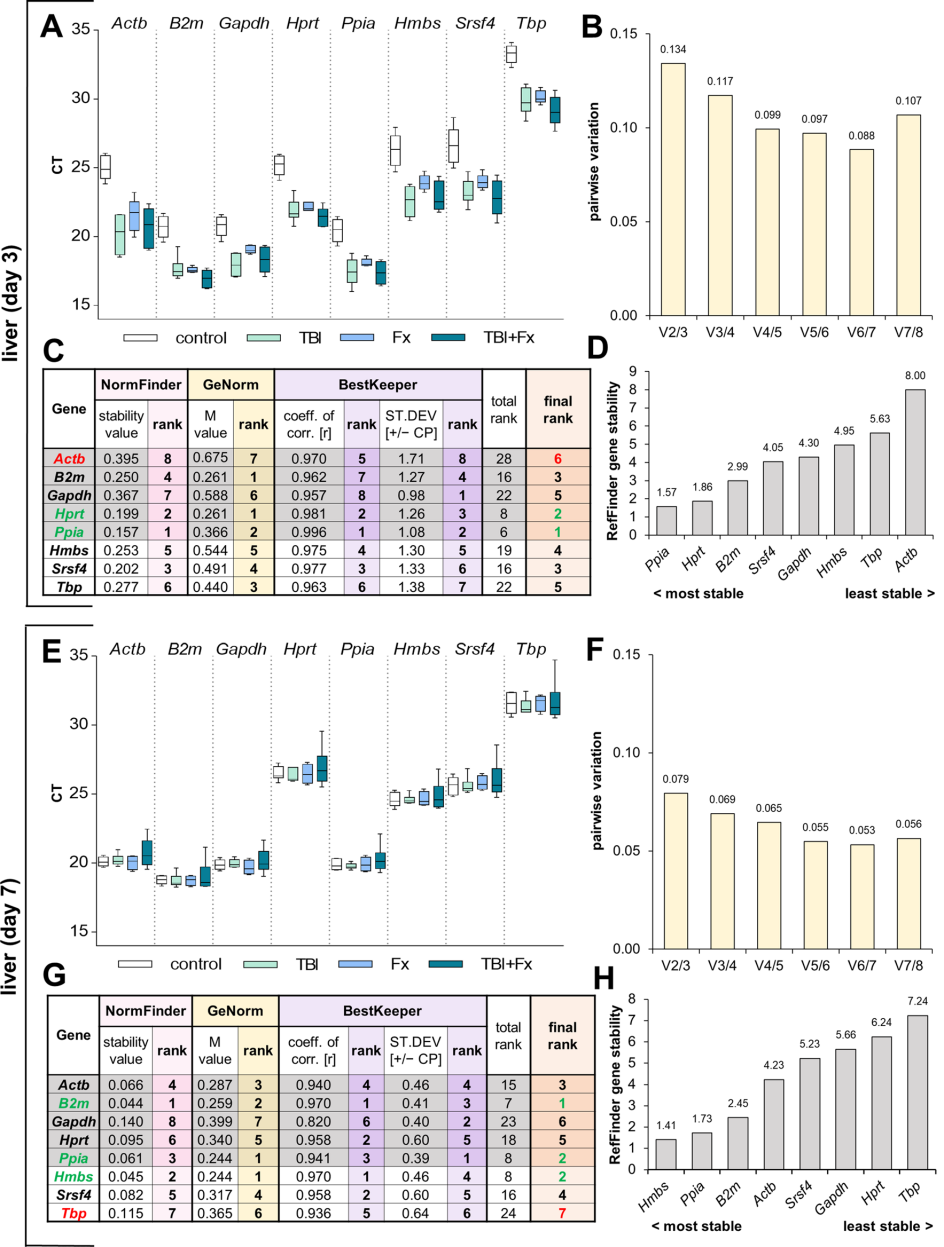
Reference gene expression stability analysis in liver tissue at three (**A**–**D**) and seven days (**E**–**H**) following surgery. (**A**/**E**) Distribution of threshold cycle (Ct) values for each group (Fx = fracture; TBI = traumatic brain injury; TBI + Fx = combined injury) of all candidate reference genes tested. (**B**/**F**) Pairwise variation (V_n/n+1_) analysis by GeNorm, to determine the optimal number of reference genes for normalisation. (**C**/**G**) Stability analysis by NormFinder (stability value: sv), GeNorm (M value) and BestKeeper (coefficient of correlation: r, standard deviation: SD), with a sum displayed in the final rank (green = most stable, red = least stable). (**D**/**H**) Overall expression stability confirmation of RefFinder, which combines NormFinder, GeNorm, BestKeeper and the comparative delta-Ct method.

Another secondary organ analysed was the liver, as altered liver metabolism is a core element of posttraumatic response^23,24^. In liver tissue at d3 after trauma high gene expression (low Ct) of *Ppia* and *B2m* was observed, while *Tbp* showed the lowest expression (Fig. 6A). NormFinder revealed the highest stability for *Ppia* (sv = 0.157) and *Hprt* (sv = 0.199), as well as great variability of *Actb* (sv = 0.395). GeNorm calculated the best stability for *Hprt* and *B2m* (M = 0.261), followed by *Ppia* (Fig. 6C). Among all housekeeping genes tested, *Actb* (M = 0.675), *Gapdh* (M = 0.588) and *Hmbs* (M = 0.544) did not reach the threshold of suitability during the GeNorm analysis. GeNorm also revealed that the combination of two genes (V_2/3_ = 0.134) is sufficient for housekeeping in the liver tissue at d3 (Fig. 6B). BestKeeper identified the best coefficient of correlation for *Ppia* (r = 0.996) and *Hprt* (r = 0.981) with a low SD. Again, our final rank as well as RefFinder confirmed *Ppia* and *Hprt* as the best reference gene combination, while *B2m* was ranked third at d3 after trauma in the liver (Fig. 6C,D).

At d7 after trauma in liver tissue (Fig. 6E–H), NormFinder revealed a low stability value for all investigated genes, ranking *B2m* first, *Hmbs* second and *Ppia* third (Fig. 6G). GeNorm identified *Ppia* and *Hmbs* as the best housekeeping gene combination. Although BestKeeper calculated the highest coefficient of correlation for *B2m* and *Hmbs*, *Ppia* still offered a high coefficient of correlation with the lowest SD. Our final ranking and RefFinder both confirmed *B2m*, *Ppia* and *Hmbs* as the best reference gene combination in liver at d7 (Fig. 6G,H).

Besides the secondary organs WAT and liver, our candidate reference genes were additionally evaluated in the metabolic- and immuno-active tissues muscle and spleen (see Supplementary Fig. S2 online). In both tissues, *Ppia* was once again identified as the most stable reference gene.

### Target gene analysis

**Figure 7:**
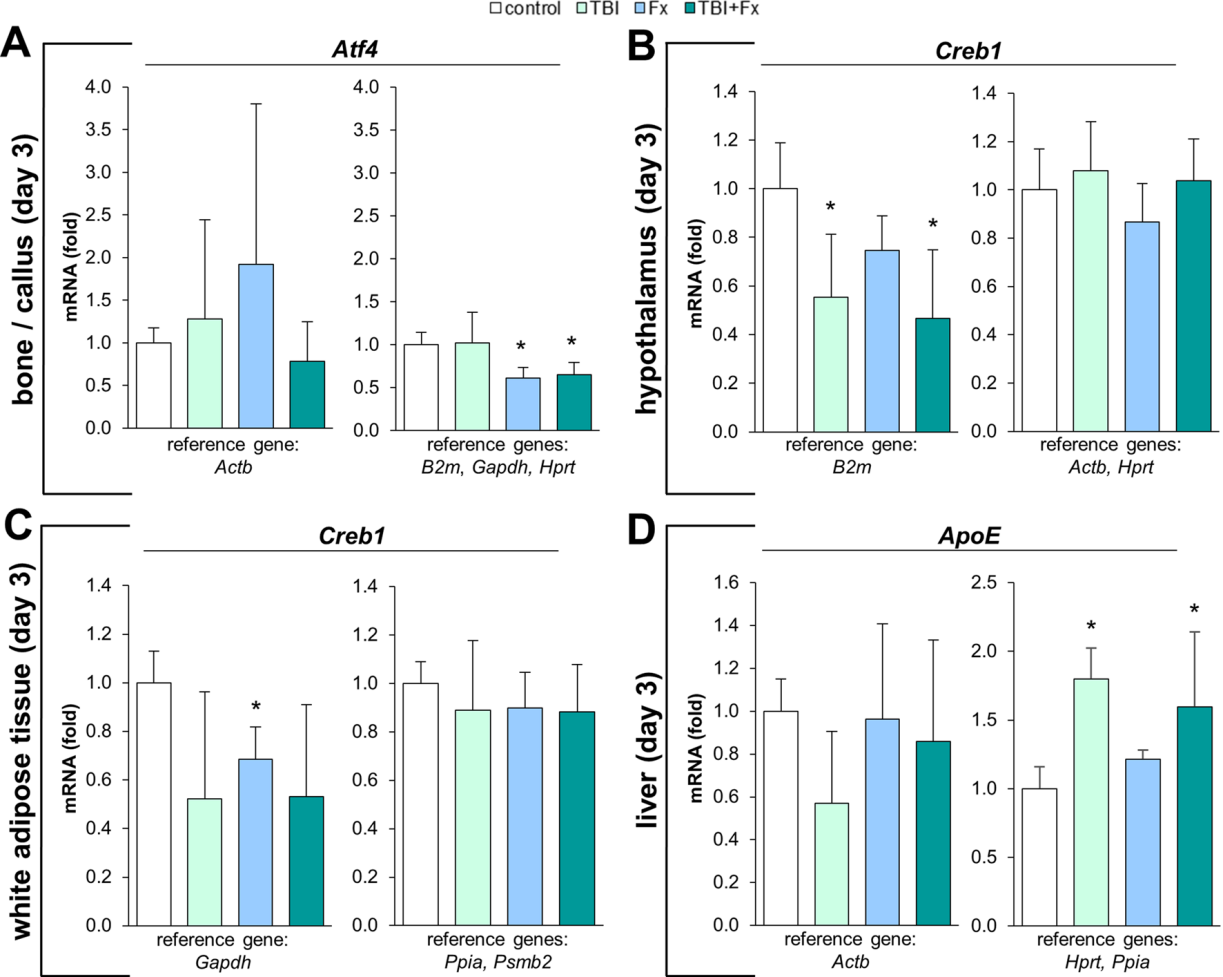
Target genes normalised to strongly regulated (left graph) as well as the most suitable reference genes (right graph) in (**A**) bone/callus, (**B**) hypothalamus, (**C**) white adipose tissue and (**D**) liver at three days (d3) following surgery (Fx = fracture; TBI = traumatic brain injury; TBI + Fx = combined injury). *p < 0.05 Mann–Whitney U test vs. control.

To illustrate the effect of the reference gene analysis conducted, we normalised trauma relevant target genes (Table 2)^31–33^ to strongly regulated as well as to the most suitable reference genes identified (Fig. 7A–D). In bone/callus (d3), the normalisation of the target gene *Atf4* to *Actb* resulted in a large variance with no informative value (Fig. 7A), whereas the use of *B2m*, *Gapdh* and *Hprt* disclosed a significant lower expression of *Atf4* in the fracture callus (groups: Fx and TBI + Fx) compared to the intact bone (groups: control and TBI). As *B2m* expression is strongly modulated in the injured hypothalamus, an artificial *Creb1* regulation was observed when applying *B2m* for housekeeping in the hypothalamus at d3. However, using the recommended reference gene combination of *Hprt* and *Actb*, a constant *Creb1* expression across all groups was revealed (Fig. 7B). In WAT (d3), *Creb1* seemed to be significantly regulated following fracture, when normalised to *Gapdh*. However, when applying the most suitable reference gene combination *Ppia* and *Psmb2*, the regulation was no longer valid (Fig. 7C). In liver (d3), *ApoE* showed no distinct regulation, after being normalised to the most unstable candidate *Actb*. When using the most suitable reference gene combination of *Ppia* and *Hprt* however, a significant regulation was revealed (Fig. 7D).

**Table 2:** qPCR primer set (murine) of all target genes tested.

Symbol	Gene name	Protein function	Primer sequence (F: 5′-3′/R: 3′-5′)
*ApoE*	Apolipoprotein E	Major apoprotein of the chylomicron, essential for the normal catabolism of lipoprotein constituents with triglycerides	F: TGGTGTTCAAAGACAATTTTTCCCT R: CCACTCGAGCTGATCTGTCAC
*Atf4*	Activating transcription factor 4	Transcription factor, also characterised as the cAMP-response element binding protein 2 (Creb2)	F: CACTGGCGAGTGTAAGGAGC R: TTCTTCCCCCTTGCCTTACG
*Creb1*	cAMP responsive element binding protein 1	Phosphorylation-dependent transcription factor	F: CCCTGCCATCACCACTGTAA R: GGTTAATGTCTGCAGGCCCT

In summary, these examples strongly underline that the application of suitable reference genes reduces false positive results (Fig. 7B,C) and strengthens relevant regulations in gene expression (Fig. 7A,D).

## Discussion

The choice of adequate reference genes for qRT-PCR normalisation is essential to detect target gene regulation, especially if the expression alteration is smaller than two fold^34,35^. Our analysis provides recommendations for the use of reference genes in a murine multiple-trauma model (Table 3). In primarily traumatised organs, housekeeping appears to be much more complicated than in secondary organs, particularly when comparing injured and intact tissues. Therefore, an individual evaluation for each condition and time point is required, which was also concluded by previous studies focusing on isolated traumatic brain injury^20,36^. For the comparison of gene expression in the intact bone and fracture callus we suggest the use of *Gapdh, Hprt* and *B2m* at three days (d3) as well as *Ppia* and *Hprt* at seven days (d7) following trauma. Although *Gapdh*^37,38^ and *Actb*^39^ were often used for housekeeping in bone studies, we recommend at least two reference genes in order to allow adequate normalisation for such a complex and multicellular organ under the challenging circumstances of trauma^34,40^.

**Table 3:** Summary of key findings and recommendations for reference genes in analysed tissues.

	Days post trauma	Reference genes recommended
**Primary organs**		
Bone/callus	d3	*Hprt* + *Gapdh* + *B2m*
	d7	*Hprt* + *Ppia*
Hypothalamus	d3	*Hprt* + *Actb*
	d7	*Hprt* + *Gapdh*
**Secondary organs**		
White adipose tissue (WAT)	d3	*Ppia* + *Psmb2*
	d7	*Ppia* + *Psmb2*
Liver	d3	*Ppia* + *B2m/Hprt*
	d7	*Ppia* + *B2m/Hmbs*
Muscle	d3	*Ppia* + *Hprt*
Spleen	d3	*Ppia* + *Hprt*

As in the hypothalamus the results of the reference gene analysis differ between d3 and d7 after trauma, no general guidance for this primary organ can be provided. Therefore, analogue to the bone/callus, we suggest an individual evaluation of the time point analysed in primarily traumatised organs to identify the most suitable housekeeping. At both time points *Hprt*, which has previously been recommended for isolated TBI and ischemic stroke models^22,41^, showed a high expression stability in the hypothalamus. Hprt, a hypoxanthine-phosphoribosyltransferase (Table 1), is essential for the synthesis of purine nucleotides^42^. The mRNA expression of *Hprt* seems to stay unaffected by the induction of TBI. In contrast, the expression of *B2m* was strongly regulated following TBI. Beta-2-microglobulin, part of the major histocompatibility complex (MHC), is directly involved in the immune response of the injured brain and therefore inappropriate for housekeeping^41,43^. As gene expression following TBI strongly depends on the trauma incidence and time point of healing, in line with previous studies^20,36^, we strongly recommend an individual assessment of the reference genes for each study condition and time point analysed prior to target gene normalisation. Nonetheless, the application of different reference genes at continuative time points allows the analysis of time-dependent target gene expression, if the data is continuously normalised to the same control group. In studies primarily focussing on time-dependent changes of gene expression in primary organs following a specific trauma, the method of gene expression analysis needs to be adapted. Therefore, the evaluation of suitable reference genes is suggestively performed analysing the CT values of a specific trauma throughout all time points.

While the reference gene evaluation in primarily traumatised organs appears to be very complex, in secondary organs, i.e. white adipose tissue (WAT), liver, muscle and spleen, *Ppia* expression remained continuously stable following isolated and combined trauma. The enzyme Ppia, also called cyclophilin A, catalyses the cis–trans isomerisation of proline imidic peptide bonds thereby accelerating protein folding^44^. In line with our results, the reference gene *Ppia* previously showed high stability in adipose tissue and liver even under challenging metabolic conditions^45,46^.

In WAT at d3 and d7 following trauma, our analysis suggests the additional use of the reference gene *Psmb2* along with *Ppia*. The encoded protein of *Psmb2* is part of the proteasome complex that controls protein degradation^47^. The application of *Psmb2* has been previously approved for housekeeping in brown adipose tissue^48^. In liver at d3 and d7, our results suggest the additional use of *B2m*, which was already approved as reference gene by Gong et al*.*^45^. Since reference gene evaluation in secondary organs is indispensable but unfortunately uncommon^49–51^, we suggest the use of *Ppia* in combination with a second reference gene as a simple guideline. However, it has to be pointed out that our study is limited to specific types of trauma induced in young female C57Bl/6J mice. Despite the high degree of expression stability of *Ppia*, further investigations are recommended to verify *Ppia* as a suitable reference gene in secondary organs of other trauma mouse models.

In summary, our work raises awareness for the precise evaluation of the reference genes used in primary organs and points to *Hprt* as a stable housekeeper. Additionally, we provide a basic guideline for the gene expression analysis in secondary organs of a murine combined trauma model: Our study revealed *Ppia* as the most suitable reference gene in white adipose tissue, liver, muscle and spleen, which in combination with a second reference gene ensures the reliability of the normalised data. This study provides the basis for reliable and reproducible gene expression analysis for future biomedical research, to elucidate the pathophysiological mechanisms underlying post traumatic alterations with the aim to enhance specific therapy development in the field of regenerative trauma surgery.

## Methods

### Animals

Female C57Bl/6J, referred to as wildtype (WT) mice, were purchased from Charles River and maintained following the regulations of animal protection and care at the Research facilities for experimental medicine (FEM, Charité–Universitätsmedizin Berlin). Upon arrival, animals were kept under standard controlled conditions: temperature 20 ± 2 °C, 12 h light/dark cycle, water ad libitum and standard diet. All experimental procedures were approved by the local legal representative animal rights protection authorities (Landesamt für Gesundheit und Soziales Berlin, G0009/12) and performed according to the policies and principles established by the national institute of health guide for care and use of laboratory animals as well as the Welfare Act (Federal Law Gazett I, p.1094).

### Trauma model

The murine multitrauma model was previously described^52^. For this study, a total number of 48 12-week old female mice (body weight: 20–25 g) were randomly assigned to the following groups (n = 6 for each group and time point): control, isolated fracture (Fx) and traumatic brain injury (TBI) as well as combined injury (TBI + Fx). Fracture was induced conducting a standardised mid-diaphyseal femoral osteotomy of the left limb. Therefore, an anterolateral skin incision was performed following an imaginal line from knee to hip joint for approximately 2 cm. Once the fascia lata was opened, the *Musculus vastus lateralis* and *Musculus biceps femoris* were bluntly dissected until the femoral bone was exposed. Now the external fixator (RISystem) was placed to drill the orthogonal pin holes by hand perpendicular to the longitudinal axis of the femur along the cortical surface. With the first pin hole just proximal of the distal metaphysis, the external fixator was screwed strictly parallel to the femur, allowing a rigid fixation. With the external fixator in place, a 0.70 mm osteotomy of was performed, applying a Gigli wire saw (RISystem) between the two middle pins. Throughout the whole procedure, the sciatic nerve was spared and its function postoperatively tested.

TBI was induced applying a standardised controlled cortical impact model (CCI) to the parietotemporal cortex. Therefore, the neurocranium was positioned and fixed in a stereotactic device, allowing a skin incision sagittal and temporal on the left side of the skull. After the *Musculus temporalis* was mobilised and the cranial bone exposed, a 7 × 7 mm temporoparietal craniotomy was microsurgically performed, while ensuring an entirely intact dura mater. Now the actual TBI was caused, applying a stereotactic computer controlled pneumatically driven bolt (3 mm flat, 45-degree angle, impact velocity of 3.5 m/s, 0.25 mm penetration depth, contact duration of 0.15 s). After the impact, the preserved piece of skull was repositioned and adapted using dental cement to suppress cranial bone healing. In the combined injury group, TBI was induced first, which was followed by fracture. All wounds were closed with Ethilon 6.0 suture. Each trauma surgery consisted of perioperative anesthesia and antibiotic prophylaxis as well as postoperative analgesia and monitoring^52^.

Three (d3) and seven days (d7) after surgery directly (primary organs), as well as indirectly trauma-affected organs (secondary organs) were obtained, snap frozen in liquid nitrogen and stored at −80 °C. Primary organs include the intact femur/fracture callus and the hypothalamus, whereas secondary organs include white adipose tissue (WAT), liver, spleen and muscle. The fracture callus was gathered between the middle pins of the external fixator (groups: Fx, TBI + Fx), with an extraction of the intact femur accordingly (groups: control, TBI). The hypothalamus was dissected and sampled macroscopically after decapitation. For white adipose tissue a gonadal fat pad, for muscle the intact *Musculus quadriceps femoris* and for liver/spleen a 0.1 cm^3^ part of the organ was obtained for RNA extraction.

### RNA extraction and cDNA synthesis

Frozen tissues were homogenised in TRIzol utilising an UltraTurrax (Sigma Aldrich) and isolated with the RNeasy mini Kit (Qiagen) combined with DNase I (Thermo Fisher) treatment. RNA concentrations were determined and purity monitored (A260:A280 ratio of 1.9–2.1) using a NanoPhotometer P360 (Implen GmbH). A tissue-dependent volume of 0.5–5 µg RNA was reverse transcribed to complementary DNA (cDNA) applying the RevertAid First Strand cDNA Synthesis Kit (ThermoFisher) and finally stored at −20 °C.

### qRT-PCR

Quantitative real-time PCR (qRT-PCR) was performed within a 384 well-plate in a 7900HT Fast Real-Time PCR System employing the software SDS v2.4. (Applied Biosystems). Power SYBR Green PCR Master Mix (Sigma Aldrich) with 2 ng cDNA per well were run at 60° annealing temperature. For each run SDS v2.4. calculated the threshold cycle (Ct), as well as the melting curve, which confirmed PCR product specificity.

The murine qRT-PCR primers of the candidate reference (Table 1) and target genes (Table 2) were individually designed using the GRCm38.p6 C57BL/6J reference genome and employing the software Primer3 (https://bioinfo.ut.ee/primer3-0.4.0/), spanning at least two exons with a large intron in between to avoid amplifications from genomic DNA. The primers were purchased from Eurofins Genomics GmbH. While the reference genes *Actb, B2m, Gapdh*, *Hprt* and *Ppia* were analysed in all tissue, *Hmbs*, *Psmb2*, *Rplp0*, *Srfs4* and *Tbp* were applied tissue-specific. For the finally analysed target genes, the expression was normalised per individual reference genes by the delta-delta Ct method^53^.

### Expression stability analysis

The candidate reference genes (Table 1) were examined for their expression stability, using the following Microsoft Excel-based algorithms: NormFinder^54^, GeNorm^28^ and BestKeeper^55^. Once all individually calculated ranks were summed to identify the “final rank”, the result was confirmed applying the web tool RefFinder (https://www.heartcure.com.au/reffinder/).

### NormFinder analysis

NormFinder incorporates the inter- and intra-group variances to determine the gene expression stability value (sv)^54^. The reference gene scoring the lowest sv is considered the most stable expressed. Finally, the algorithm ranks all assessed candidate reference genes based on their sv.

### GeNorm analysis

The GeNorm algorithm defines the most suitable reference gene pair whose expression ratio is least affected by external factors of varying samples and experimental conditions^28^. Therefore, GeNorm calculates the stability value (M), which is based on the pairwise variation of one gene compared to all other assessed candidate reference genes, with a stepwise exclusion of the highest M value. As a result, the candidates with the lowest M value are considered the reference genes with the highest gene expression stability. The GeNorm file suggests a threshold of 1.5 for suitable reference genes, however, when dealing with homogeneous sample panels the threshold can be set to M < 0.5^27^. Additionally, GeNorm determines the minimal number of reference genes necessary to allow a sufficient gene expression normalisation. The accuracy of normalisation will not significantly improve by adding an additional reference gene, if the pairwise variation is below the cut-off V_n/n+1_ < 0.15^28^.

### BestKeeper analysis

BestKeeper determines the coefficient of correlation according to the BestKeeper index, which represents the geometric means of the assessed candidate reference gene Ct values^55^. The algorithm calculates the coefficient of correlation (r) and a standard derivation (SD) of the crossing point (Cp), which is analogue to the Ct. Reference genes with the highest r and the lowest SD are considered the most stable expressed.

### RefFinder analysis

RefFinder evaluates the assessed reference genes according to the geometric mean of the ranks identified by the previously described algorithms NormFinder, GeNorm and BestKeeper as well as the comparative delta-Ct method^56^. Therefore, the RefFinder algorithm provides a valid overall confirmation of the expression stability analyses.

### Statistical analysis

After testing for normal distribution (Shapiro–Wilk test), target gene expression was analysed applying the non-parametric Mann–Whitney U test (GraphPad Prism 6.0). All data are reported as mean ± standard deviation (SD). P < 0.05 was considered statistically significant (*). Microsoft Excel and GraphPad Prism 6.0 software (GraphPad Software Inc.) for Windows were used to graph the data.

## Supplementary information

Supplementary Information.

## Data Availability

The datasets analysed during the current study are available from the corresponding author on reasonable request.

## References

[1] Demetriades D (2004). Trauma fatalities: Time and location of hospital deaths. J. Am. Coll. Surg..

[2] Probst C (2009). 30 years of polytrauma care: An analysis of the change in strategies and results of 4849 cases treated at a single institution. Injury.

[3] Søreide K (2009). Epidemiology of major trauma. Br. J. Surg..

[4] 4World-Health-Organization. *Global Health Estimates 2016: Deaths by Cause, Age, Sex, by Country and by Region 2000–2016* (2018).

[5] Keel M, Trentz O (2005). Pathophysiology of polytrauma. Injury.

[6] Flohé SB, Flohé S, Schade FU (2008). Invited review: deterioration of the immune system after trauma: Signals and cellular mechanisms. Innate Immun..

[7] Lord JM (2014). The systemic immune response to trauma: An overview of pathophysiology and treatment. Lancet.

[8] Xiao W (2011). A genomic storm in critically injured humans. J. Exp. Med..

[9] Weckbach S (2012). A new experimental polytrauma model in rats: Molecular characterization of the early inflammatory response. Mediators Inflamm..

[10] Rittirsch D (2015). Improvement of prognostic performance in severely injured patients by integrated clinico-transcriptomics: A translational approach. Crit. Care.

[11] Tremoleda JL, Watts SA, Reynolds PS, Thiemermann C, Brohi K (2017). Modeling acute traumatic hemorrhagic shock injury: Challenges and guidelines for preclinical studies. Shock.

[12] Helmy A, De Simoni MG, Guilfoyle MR, Carpenter KL, Hutchinson PJ (2011). Cytokines and innate inflammation in the pathogenesis of human traumatic brain injury. Prog. Neurobiol..

[13] Torrance HD (2015). Association between gene expression biomarkers of immunosuppression and blood transfusion in severely injured polytrauma patients. Ann. Surg..

[14] Yang L (2016). Bone fracture enhances trauma brain injury. Scand. J. Immunol..

[15] Sun M (2017). Treatment with an interleukin-1 receptor antagonist mitigates neuroinflammation and brain damage after polytrauma. Brain Behav. Immun..

[16] Huggett J, Dheda K, Bustin S, Zumla A (2005). Real-time RT-PCR normalisation; strategies and considerations. Genes Immun..

[17] Thellin O (1999). Housekeeping genes as internal standards: Use and limits. J. Biotechnol..

[18] Barber RD, Harmer DW, Coleman RA, Clark BJ (2005). GAPDH as a housekeeping gene: Analysis of GAPDH mRNA expression in a panel of 72 human tissues. Physiol. Genomics.

[19] Montero-Melendez T, Perretti M (2014). Gapdh gene expression is modulated by inflammatory arthritis and is not suitable for qPCR normalization. Inflammation.

[20] Thal SC, Wyschkon S, Pieter D, Engelhard K, Werner C (2008). Selection of endogenous control genes for normalization of gene expression analysis after experimental brain trauma in mice. J. Neurotrauma.

[21] Melgar-Rojas P, Alvarado JC, Fuentes-Santamaría V, Gabaldón-Ull MC, Juiz JM (2015). Validation of reference genes for RT-qPCR analysis in noise-induced hearing loss: A study in Wistar rat. PLoS ONE.

[22] 22Kang, Y., Wu, Z., Cai & Lu, B. Evaluation of reference genes for gene expression studies in mouse and N2a cell ischemic stroke models using quantitative real-time PCR. *BMC Neurosci.* **19**, 3, 10.1186/s12868-018-0403-6 (2018). 10.1186/s12868-018-0403-6 PMC5795833 29390963

[23] Rege SD (2019). Brain trauma disrupts hepatic lipid metabolism: Blame it on fructose?. Mol. Nutr. Food Res..

[24] Şimşek T, Şimşek HU, Cantürk NZ (2014). Response to trauma and metabolic changes: Posttraumatic metabolism. Ulus Cerrahi Derg.

[25] 25Li, M. & Sirko, S. Traumatic brain injury: At the crossroads of neuropathology and common metabolic endocrinopathies. *J. Clin. Med.* **7**, 10.3390/jcm7030059 (2018). 10.3390/jcm7030059 PMC5867585 29538298

[26] Rasouli J, Lekhraj R, Ozbalik M, Lalezari P, Casper D (2011). Brain-spleen inflammatory coupling: A literature review. Einstein J. Biol. Med..

[27] Hellemans J, Mortier G, De Paepe A, Speleman F, Vandesompele J (2007). qBase relative quantification framework and software for management and automated analysis of real-time quantitative PCR data. Genome Biol..

[28] 28Vandesompele, J. *et al.* Accurate normalization of real-time quantitative RT-PCR data by geometric averaging of multiple internal control genes. *Genome Biol.* **3**, RESEARCH0034, 10.1186/gb-2002-3-7-research0034 (2002). 10.1186/gb-2002-3-7-research0034 PMC126239 12184808

[29] Ciechanowska A (2020). Changes in macrophage inflammatory protein-1 (MIP-1) family members expression induced by traumatic brain injury in mice. Immunobiology.

[30] Förstner P (2018). Neuroinflammation after traumatic brain injury is enhanced in activating transcription factor 3 mutant mice. J. Neurotrauma.

[31] Wen AY, Sakamoto KM, Miller LS (2010). The role of the transcription factor CREB in immune function. J. Immunol..

[32] 32Li, L. *et al.* The association between apolipoprotein E and functional outcome after traumatic brain injury: A meta-analysis. *Medicine (Baltimore)* **94**, e2028, 10.1097/MD.0000000000002028 (2015). 10.1097/MD.0000000000002028 PMC4652820 26579811

[33] Makowski AJ (2014). The loss of activating transcription factor 4 (ATF4) reduces bone toughness and fracture toughness. Bone.

[34] Yang X (2012). Bone to pick: The importance of evaluating reference genes for RT-qPCR quantification of gene expression in craniosynostosis and bone-related tissues and cells. BMC Res. Notes.

[35] Ramhøj L, Axelstad M, Svingen T (2019). Validation of endogenous reference genes in rat cerebral cortex for RT-qPCR analyses in developmental toxicity studies. PeerJ.

[36] Harris JL, Reeves TM, Phillips LL (2009). Injury modality, survival interval, and sample region are critical determinants of qRT-PCR reference gene selection during long-term recovery from brain trauma. J. Neurotrauma.

[37] Khan SN (2008). Identification of novel gene expression in healing fracture callus tissue by DNA microarray. HSS J..

[38] Yuan C, Cai J (2017). Time-series expression profile analysis of fracture healing in young and old mice. Mol. Med. Rep..

[39] Bais M (2009). Transcriptional analysis of fracture healing and the induction of embryonic stem cell-related genes. PLoS ONE.

[40] Schulze F (2017). A tissue-based approach to selection of reference genes for quantitative real-time PCR in a sheep osteoporosis model. BMC Genomics.

[41] Timaru-Kast R, Herbig EL, Luh C, Engelhard K, Thal SC (2015). Influence of age on cerebral housekeeping gene expression for normalization of quantitative polymerase chain reaction after acute brain injury in mice. J. Neurotrauma.

[42] 42Ansari, M. Y., Dikhit, M. R., Sahoo, G. C. & Das, P. Comparative modeling of HGPRT enzyme of *L. donovani* and binding affinities of different analogs of GMP. *Int. J. Biol. Macromol.* **50**, 637–649, 10.1016/j.ijbiomac.2012.01.010 (2012). 10.1016/j.ijbiomac.2012.01.010 22327112

[43] Rhinn H (2008). Housekeeping while brain's storming Validation of normalizing factors for gene expression studies in a murine model of traumatic brain injury. BMC Mol. Biol..

[44] Hoffmann H, Schiene-Fischer C (2014). Functional aspects of extracellular cyclophilins. Biol. Chem..

[45] Gong H (2016). Evaluation of candidate reference genes for RT-qPCR studies in three metabolism related tissues of mice after caloric restriction. Sci. Rep..

[46] Nakao R, Okauchi H, Hashimoto C, Wada N, Oishi K (2017). Determination of reference genes that are independent of feeding rhythms for circadian studies of mouse metabolic tissues. Mol. Genet. Metab..

[47] Tomko RJ, Hochstrasser M (2013). Molecular architecture and assembly of the eukaryotic proteasome. Annu. Rev. Biochem..

[48] Taube M (2015). Evaluation of reference genes for gene expression studies in human brown adipose tissue. Adipocyte.

[49] Barkhausen T, Hildebrand F, Krettek C, van Griensven M (2009). DHEA-dependent and organ-specific regulation of TNF-alpha mRNA expression in a murine polymicrobial sepsis and trauma model. Crit. Care.

[50] 50Dickens, A. M. *et al.* Astrocyte-shed extracellular vesicles regulate the peripheral leukocyte response to inflammatory brain lesions. *Sci. Signal* **10**, 10.1126/scisignal.aai7696 (2017). 10.1126/scisignal.aai7696 PMC5590230 28377412

[51] Fitschen-Oestern S (2017). Hepatocytes express the antimicrobial peptide HBD-2 after multiple trauma: An experimental study in human and mice. BMC Musculoskelet. Disord..

[52] Tsitsilonis S (2015). The effect of traumatic brain injury on bone healing: an experimental study in a novel in vivo animal model. Injury.

[53] Livak KJ, Schmittgen TD (2001). Analysis of relative gene expression data using real-time quantitative PCR and the 2(-Delta Delta C(T)) method. Methods.

[54] Andersen CL, Jensen JL, Ørntoft TF (2004). Normalization of real-time quantitative reverse transcription-PCR data: A model-based variance estimation approach to identify genes suited for normalization, applied to bladder and colon cancer data sets. Cancer Res..

[55] Pfaffl MW, Tichopad A, Prgomet C, Neuvians TP (2004). Determination of stable housekeeping genes, differentially regulated target genes and sample integrity: BestKeeper–Excel-based tool using pair-wise correlations. Biotechnol. Lett..

[56] Xie F, Xiao P, Chen D, Xu L, Zhang B (2012). miRDeepFinder: A miRNA analysis tool for deep sequencing of plant small RNAs. Plant Mol. Biol..

