# Supplementary material for: Validation of reference genes for expression analysis in a murine trauma model combining traumatic brain injury and femoral fracture
Source: Sci Rep. 2020 Sep 14;10:15057. doi: 10.1038/s41598-020-71895-x (PMC7490670; doi:10.1038/s41598-020-71895-x)
Supplement: Supplementary file 1 — Supplementary Information. [file 41598_2020_71895_MOESM1_ESM.pdf]

Validation of reference genes for expression analysis in a murine trauma model combining traumatic brain injury and femoral fracture

AUTHORS: Ellen Otto [1,2)]* AND Paul Köhli [1,2)]*, Jessika Appelt [1,2)], Stefanie Menzel [1,2)], Melanie Fuchs [1,2)], Alina Bahn [1,2)], Frank Graef [1,2)], Georg N. Duda [1)], Serafeim Tsitsilonis[1,2)], Johannes Keller [1,3)] and Denise Jahn [1,2)]

AFFILIATIONS:
[1)] Julius Wolff Institute for Biomechanics and Musculoskeletal Regeneration, Charité-Universitätsmedizin Berlin, 13353 Berlin, Germany.
[2)] Center for Musculoskeletal Surgery, Charité - Universitätsmedizin Berlin, 13353 Berlin, Germany.
[3)] Department of Trauma and Orthopedic Surgery, University Medical Center Hamburg-Eppendorf, Hamburg 20246, Germany.

* These authors contributed equally to this work.

Supplementary information

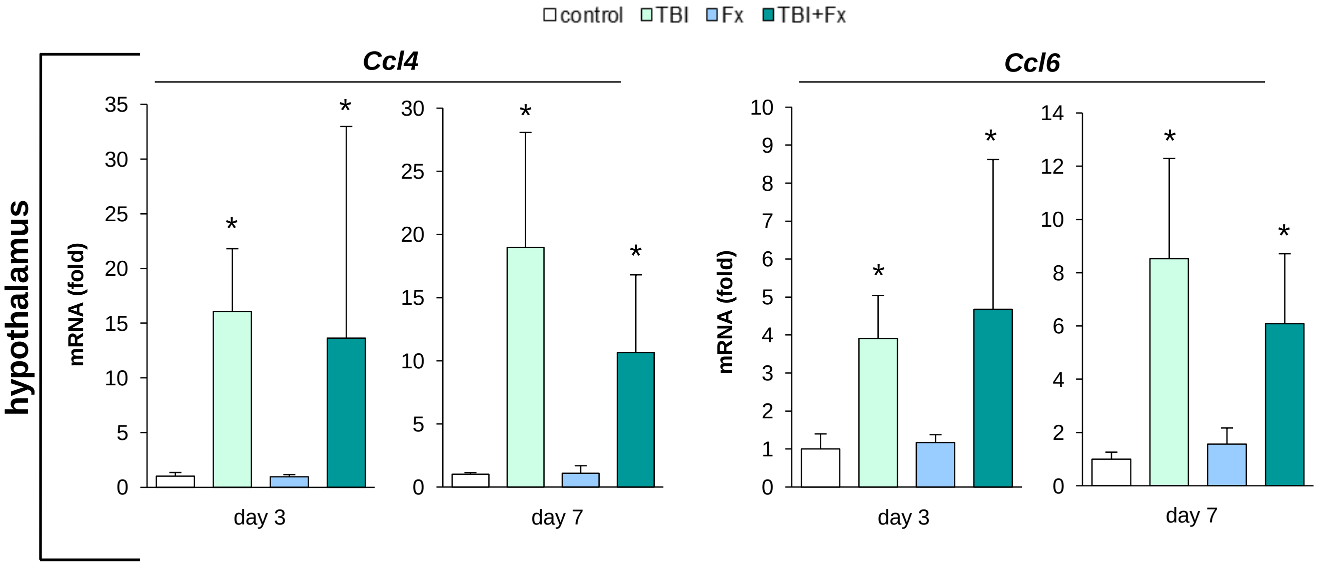

Supplementary figure S1. mRNA expression of *Ccl4* and *Ccl6* in the hypothalamus confirming neuroinflammation following TBI. *p<0.05 Mann-Whitney-U-Test vs. control

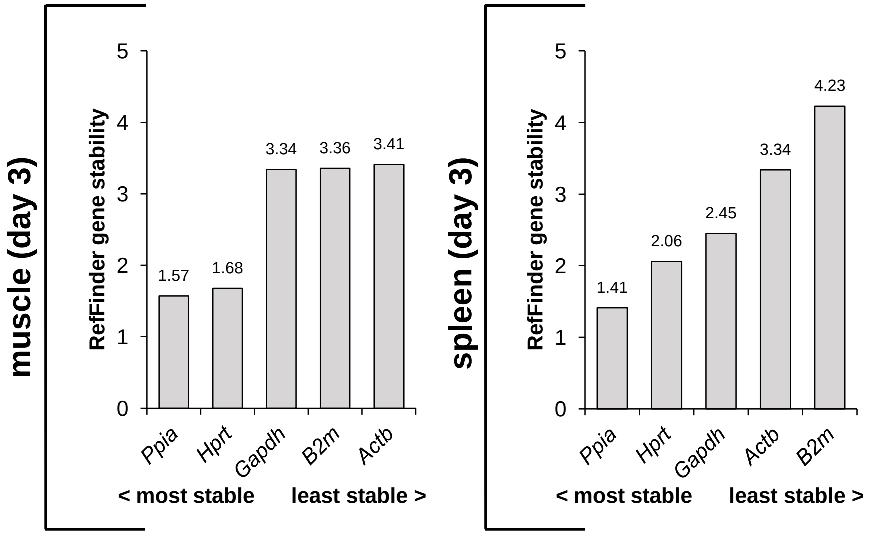

Supplementary figure S2. Overall expression stability confirmation of RefFinder, which combines NormFinder, GeNorm, BestKeeper and the comparative delta-Ct method, in muscle and spleen tissue at three days (d3) following surgery.
